# Does full HPV genotyping perform similarly well in clinician-collected cervical samples and self-collected vaginal samples when using the CLART HPV4S assay?

**DOI:** 10.1186/s12905-023-02215-4

**Published:** 2023-02-23

**Authors:** Jannie Villekjær Solnæs, Sisse Helle Njor, Mette Tranberg

**Affiliations:** 1 Faculty of Health, Aarhus University, Vennelyst Boulevard 4, 8000 Aarhus C, Denmark; 2 University Research Clinic for Cancer Screening, Department of Public Health Programmes, Randers Regional Hospital, Skovlyvej 15, 8930 Randers NØ, Denmark; 3 Department of Clinical Medicine, Aarhus University, Incuba Skejby, Building 2, Palle Juul-Jensens Boulevard 82, 8200 Aarhus N, Denmark

**Keywords:** Human papillomavirus, Self-sampling, Cervical cancer screening, Genotyping, Triage

## Abstract

**Background:**

Studies comparing self-collected vaginal samples with clinician-collected cervical samples with respect to high-risk human papillomavirus (HPV) detection and genotype agreement based on clinically validated full HPV genotype assays (e.g. the CLART HPV4S) are limited. This study compared the two types of samples using the CLART assay with respect to HPV detection and genotype agreement in a referral population.

**Methods:**

A total of 212 women aged 30–59 years and diagnosed with atypical squamous cells of undetermined significance (ASC-US) within the Danish cervical cancer screening programme had a cervical sample taken at their general practitioner. Afterwards, the women took a vaginal sample with the Evalyn Brush device at home. The paired samples were HPV-tested with the full genotyping CLART HPV4S assay. Histological results, i.e. cervical intraepithelial neoplasia of grade 2 or worse (CIN2+) were available for 14 women with HPV-positive clinician-collected samples.

**Results:**

The study found the same HPV prevalence in self-collected vaginal samples compared to clinician-collected cervical samples (19.3%, 95% CI 14.3–25.3% vs 18.4%, 95% CI 13.4–24.3%). The CLART HPV4S assay detected approximately the same number of CIN2+ cases in the self-collected vaginal samples compared to the clinician-collected cervical samples (13 vs 11 cases). Exactly the same genotypes were detected in 75% (21/28) of the HPV-positive paired samples, while at least one identical genotype was found in the remaining 25% (7/28) of the paired samples.

**Conclusions:**

The CLART HPV4S assay performed similarly well in self-collected vaginal samples as in clinician-collected cervical samples with respect to both HPV detection and genotype agreement when using the Evalyn Brush and the CLART HPV4S assay in a referral population. Although further evaluation is needed, the findings suggest that full HPV genotyping based on the CLART assay can be useful when establishing HPV genotype-specific referral strategies for women tested HPV-positive by self-sampling.

## Background

High-risk human papillomavirus (HPV) testing on self-collected vaginal samples (self-samples) has proven an effective screening method for reaching under-screened women who are reluctant to attend routine clinician-based cervical cancer screening [1, 2]. Across screening and referral populations, meta-analyses have shown that the sensitivity of HPV testing on self-samples is comparable to that of clinician-based cervical samples in terms of detecting Cervical Intraepithelial Neoplasia, CIN of grade 2 or worse (CIN2+) provided that Polymerase Chain Reaction (PCR)-based HPV DNA tests are used [1, 3]. Accordingly, several countries including Denmark have already or are in the progress of introducing vaginal self-sampling in their routine cervical cancer screening programme [2, 4–6]. As HPV tests cannot discriminate between transient HPV infections and persistent HPV infections associated with CIN2+, additional triage or risk assessment of women who are HPV positive is needed to avoid unnecessary colposcopy referrals [7]. In most countries, women with an HPV-positive self-sample regardless of HPV-genotype will be recommended to visit a clinician for additional cervical cytology triage before a possible colposcopy referral [1, 8]. However, this two-step triage approach may be associated with loss to follow-up and diagnostic delay [1]. To reduce loss to follow-up, an approach could be designed that takes the risk of underlying disease for each specific HPV-type into consideration. This approach would require the availability of full HPV genotype assays that perform equally well in clinician-collected samples and self-samples. At present, 13 HPV genotypes have been defined as "high-risk" for the development of cervical cancer [9, 10]. Accordingly, a European cohort study conducted in a referral population has demonstrated that the risk of CIN2 and CIN grade 3 and cancer (CIN3+) is genotype specific with large variations. HPV16 18, 31, and 33/58 causes the highest risk while the combination of HPV56/59/66 causes the lowest risk of disease progression and cancer [6].

Among commercially available full genotype assays, the CLART HPV4S assay (GENOMICA) is a clinically validated PCR-based HPV DNA test. It detects 14 individual oncogenic HPV genotypes [11]. Yet, to the best of our knowledge, no studies have evaluated whether CLART HPV4S performs similarly well in paired self-samples and clinician-collected cervical samples.

This study investigated whether the CLART HPV4S assay performed similarly well with respect to HPV detection and genotype agreement in paired self-samples collected at home and clinician-collected cervical samples in a referral population.

## Materials and methods

### Setting

In Denmark, cervical cancer screening is a nationwide free-of-charge population-based programme that invites women aged 23–64 years for liquid-based cervical cytology sampling at their general practitioner (GP) [12, 13]. Currently, Denmark is in the progress of transitioning from cytology-based to HPV-based screening in women aged 30 and above [4]. However, at the time of this study in 2015–2016 women aged 23–59 years were screened with cytology following the Bethesda 2001 criteria [14], while women aged 60–64 underwent primary HPV-based screening. Women aged 30–59 years who were diagnosed with ASC-US had reflex HPV triage testing performed. ASC-US/HPV-positive women were referred for colposcopy with biopsies while ASC-US/HPV-negative women were referred back to the routine screening program [12, 15]. This study was conducted in the Central Denmark Region (CDR) which covers 23% of the Danish population (1.3 million inhabitants) [16]. In CDR, all cervical cytology specimens are handled and analysed by the Department of Pathology, Randers Regional Hospital, and the COBAS 4800 HPV DNA test (Roche Diagnostics, Switzerland) is the routine test platform.

### Participants

This cross-sectional study was conducted between June 2015 and December 2016. Paired clinician-collected cervical samples and self-samples, taken at home, were obtained from women aged 30–59 years diagnosed with ASC-US within the screening programme. Exclusion criteria were pregnancy, childbirth less than three months before inclusion, and obtainment of the vaginal self-sample after colposcopy. The inclusion procedure has been described in detail in previous publications [15, 17]. In brief, eligible women received written information about the study and a consent form at the same time. The women accepting to participate contacted the investigator for oral information and returned a signed informed consent. Non-responders to the initial study invitation received a postal reminder letter within two weeks encouraging them to participate.

### Sample collection, processing and HPV testing

At screening, the women had a cervical cytology sample collected using the Cervex-Combi Brush® (Rovers Medical Devices, B.V, Oss, Netherlands) at their GP. This brush head was rinsed in 10 mL SurePath medium (BD Diagnostics, Burlington, NC) and mailed to the Department of Pathology, Randers Regional Hospital, for routine processing and testing as previously described [15, 17]. From the SurePath vial, 1 mL of the sample cell pellet was used for DNA purification on the COBAS × 480, and amplification and HPV reflex testing were performed as per routine using the COBAS z480 analyser. For this study, 100 μl of the residual purified DNA material was subsequently stored at − 80 °C before CLART testing. After cervical sampling at the GP and informed consent was obtained, a self-sampling package was mailed to the woman at her home address. The package included user instructions, a dry brush device [18] (Evalyn® Brush, Rovers Medical Devices, B. V, Oss, Netherlands) for vaginal self-sampling, and a stamped return envelope with the address of the laboratory. The women were encouraged to return the self-sample by ordinary mail on the same day as the sample was taken and before a potential colposcopy (average days from sample collection to arrival at the laboratory: 2-6 days). At the laboratory, the dry brush head was rinsed in 10 mL SurePath medium and stored overnight at 4 °C and then vortexed for 5 min. A 6.4 mL volume of the vaginal self-sample material was centrifuged at 3000 × RPM for 20 min at room temperature [15]. With the supernatant removed, the cell pellet was placed in 1 mL 25% ethanol-buffered (TRIS) and stored at − 80 °C (range: 4 days to 18 months, median: 10 months) prior to DNA purification and HPV testing using the Cobas 4800 assay. From each self-sample, 100 μl of the residual purified DNA material were stored at − 80 °C, until further CLART HPV testing. The CLART HPV4S (GENOMICA) is a PCR-based microarray assay targeting the HPV L1 region. It detects 14 individual high risk HPV genotypes: HPV16, 18, 31, 33, 35, 39, 45, 51, 52, 56, 58, 59, 66, 68; and two low risk HPV genotypes: HPV6 and 11 [11]. Amplification of a spiked Cystic Fibrosis Transmembrane conductance Regulator (CFTR) plasmid served as an internal control of the PCR process, while the internal control for human CFTR gene validates specimen quality in the sample [11]. Before the day of the CLART HPV DNA genotype testing, the residual purified DNA material from the GP-collected samples and self-samples were thawed overnight at 4 °C (after storage for 2 to 25 months, median: 12 months). In short, 5 μl aliquots of purified DNA were used for the CLART HPV4S PCR amplification. The genotyping results were interpreted and automatically analysed on the Clinical Array Reader (GENOMICA) [11]. Samples with an invalid test result defined by no human CFTR or no spiked CFTR plasmid amplification detected, were re-tested once and the second result was considered definitive. For the CLART testing, the paired cervical clinician-collected samples and self-samples belonging to the same woman were analysed in the same run to avoid between-run discrepancies. The laboratory personnel performing the HPV testing were blinded as to the HPV results of the cervical samples [15].

### Outcome measures

A sample was defined as HPV positive if at least one of these 14 genotypes was detected: HPV16, 18, 31, 33, 35, 39, 45, 51, 52, 56, 58, 59, 66, and 68. If the sample only detected HPV6 and/or HPV11, it was considered HPV negative.

To account for the low number of HPV positives, the genotypes were hierarchically subdivided into five groups based on the oncogenicity for cervical (pre-)cancer [6]: (1) HPV16/18, (2) HPV31/33/58, (3) HPV45/51/52, (4) HPV35/39/68, and (5) HPV56/59/66. Samples with multiple HPV infections were categorised using the highest risk genotype and could not occur in other categories.

Histological results were grouped using the most severe diagnosis as either ≤ CIN1 (normal tissue, CIN, CIN1) or CIN2+ (including CIN2, CIN3/adenocarcinoma in situ and carcinoma) [19]. Histological results were only available for those who tested HPV positive on the COBAS 4800 assay [15, 17]. The cervical clinician-collected sample results as well as histological results were obtained from the Danish Pathology Data Bank [20]. The results of the self-samples were retrieved from the Department of Pathology, Randers Region Hospital.

### Statistical analysis

For analysis, only women with valid HPV results from paired cervical clinician-collected samples and self-samples (paired samples) were included. HPV concordance (any type) between the paired samples was assessed using Cohen’s kappa statistics (κ) and defined as: “Poor” (κ ≤ 0.20), “Fair” (0.21 ≤ κ ≤ 0.40), “Moderate” (0.41 ≤ κ ≤ 0.60), “Good” (0.61 ≤ κ ≤ 0.80), or “Very good” (κ ≥ 0.81) [21]. The overall percentage of agreement between the paired samples was calculated as the proportion of concordant sample results divided by the total number of samples. Two-tailed McNemars test was used to test for significant differences in HPV positivity between the paired samples. Continuous variables were reported by medians and interquartile ranges (IQR) with Mann Whitney U test to test for differences. Concordance estimates (kappa and overall agreement) were reported with 95% confidence intervals (CIs). P-values ˂ 0.05 were regarded statistically significant. All statistics were calculated using STATA, version 16.1 (STATA college). A sample size calculation has been reported elsewhere [15]. All data were stored and entered in REDCap [22].

### Ethical clearance

This project was approved by The Danish Data Protection Agency (journal no.:1–10–72-69-15) and due to the use of human biological material, also by the local Ethical Committee of the CDR (journal no.: 1–16–209-15). All participants gave written informed consent to participate in the study. All methods were carried out in accordance with relevant guidelines and regulations.

## Results

### Study population

**Fig. 1:**
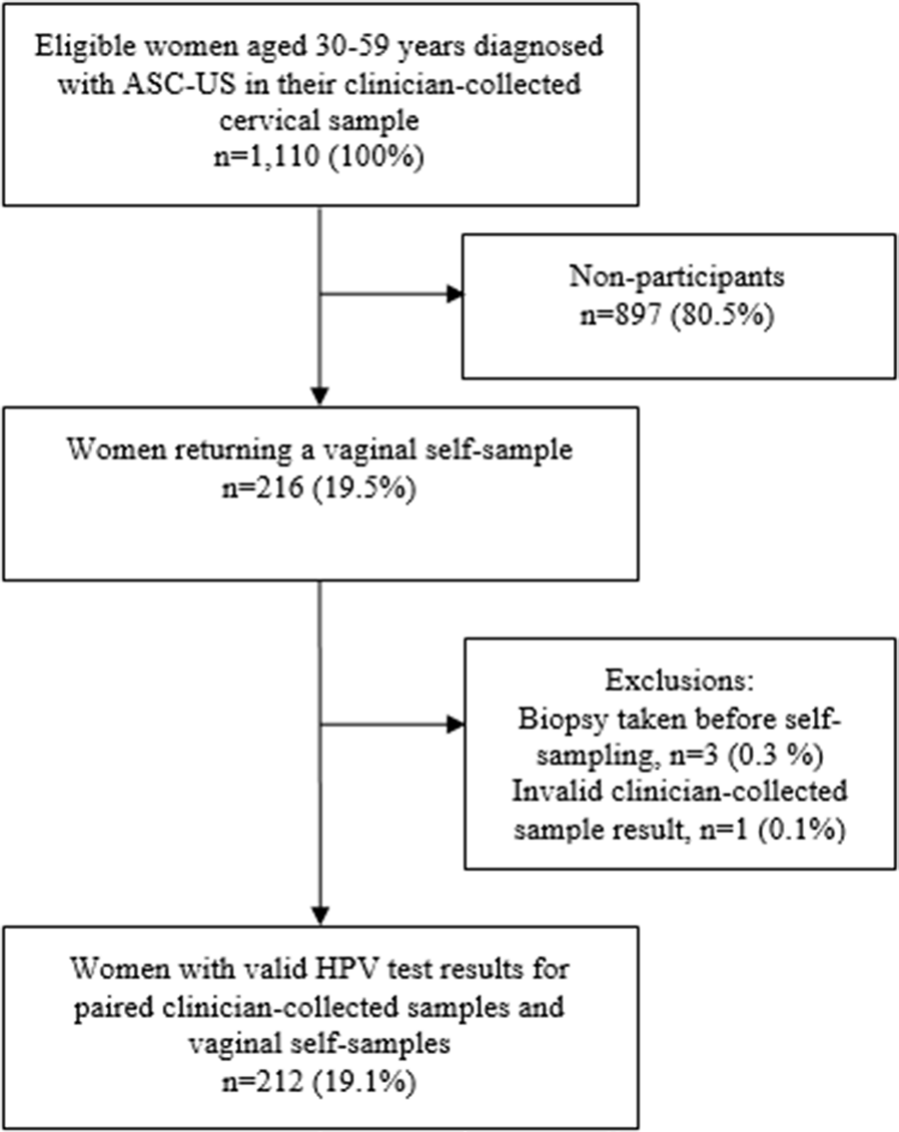
Flowchart of study population. *ASC-US* atypical squamous cells of undetermined significance, *HPV* human papillomavirus

A total of 1,110 eligible women were invited of whom 216 (19.5%) returned a self-sample (Fig. 1). Three women (0.3%) were excluded due to the self-sample being collected after the colposcopy and biopsies and 1 woman (0.1%) was excluded due to an invalid HPV test result in the clinician-collected sample, leaving 212 (19.1%) women for analysis (Fig. 1). Median age at the time of the clinician-collected samples was 44 years (IQR: 38–49 years). Median number of days between the paired samples was 43 days (IQR: 34–53 days). Histological results were available for 46 women of whom 32 had ≤ CIN1 and 14 had CIN2+.

### HPV prevalence and agreement

The HPV prevalence (any type) detected in the self-samples was similar to the prevalence in the clinician-collected samples (19.3%, 95% CI 14.3–25.3% vs 18.4%, 95% CI 13.4–24.3%, p-value 0.84) (Table 1). In total, 28 paired samples were HPV positive in both the clinician-collected sample and the self-sample. A total of 24 paired samples (11.3%) were conflicting. Out of these, 13 paired samples (6.1%) were tested HPV negative in the clinician-collected sample and HPV positive in the self-sample and 11 were tested HPV positive in the clinician-collected sample and HPV negative in the self-sample. Among the latter 11 paired samples, the median number of days between the two paired samples were significantly higher than among the 28 concordant HPV-positive paired samples (50 vs 35 days, p = 0.014) (data not shown). The detection of HPV (any type) in the clinician-collected samples and self-samples showed good concordance (κ: 0.63, 95% CI 0.50–0.77) with an overall level of agreement of 88.7% (95% CI 83.6–92.6%) (Table 1).

**Table 1:** Concordance and overall agreement for HPV detection (any type) between self-samples and clinician-collected samples (n = 212)

	Clinician-collected samples						κ^b^	Agreement	Sensitivity	Specificity
	HPV positive^a^		HPV negative		Total			(%) (95% CI)	(%) (95% CI)	(%) 95% CI)
	n	%	n	%	n	%				
Self-samples										
HPV positive^a^	28	13.2	13	6.1	41	19.3	0.63 (0.50–0.77)	88.7 (83.6–92.6)	71.8 (55.1–85.0)	92.5 (87.5–95.9)
HPV negative	11	5.2	160	75.5	171	80.7				
Total	39	18.4	173	81.6	212	100				

*HPV* human papillomavirus, *CI* confidence interval

^a^HPV positive: Genotype 16, 18, 31, 33, 35, 39, 45, 51, 52, 56, 58, 59, 66 and 68

^b^Cohen’s Kappa: “Poor” (κ ≤ 0.20), “Fair” (0.21 ≤ κ ≤ 0.40), “Moderate” (0.41 ≤ κ ≤ 0.60), “Good” (0.61 ≤ κ ≤ 0.80), or “Very good” (κ ≥ 0.81) [21]. % = Row percentage

A total of 13 CIN2+ cases were detected in the HPV-positive self-samples, whereas 11 CIN2+ cases were detected in the HPV positive clinician-collected samples.

Multiple infections with more than one genotype were observed in 31% of all HPV-positive clinician-collected samples (12/39). The corresponding percentage was 32% of all HPV-positive self-samples (13/41). Exactly the same genotypes were detected in 75% (21/28) of the paired samples that were HPV positive in both the clinician-collected sample and the self-sample, while at least one identical genotype was found in the remaining 25% (7/28) of the paired samples (data not shown).

**Fig. 2:**
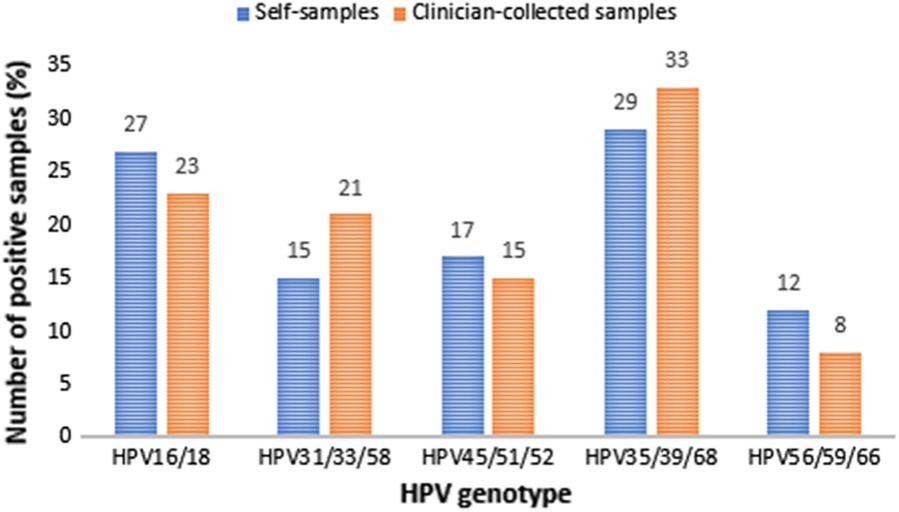
Prevalence of HPV genotype groups in HPV-positive self-samples (n = 41*) and clinician-collected samples (n = 39*). *HPV* human papillomavirus. *The difference in observations is due to higher HPV positivity in self-samples

HPV35/39/68 was the genotype group most frequently observed both in the clinician-collected samples and self-samples constituting 33% and 29% of the HPV genotypes (Fig. 2). The second most common genotype group in both samples was HPV16/18 with 23% and 27%, respectively. Using self-sampling resulted in approximately the same number of HPV16/18 cases than when clinician-collected samples were used (5.2% vs 4.3%, p-value 0.69) (Table 2). Approximately the same number of HPV31/33/58 cases were found when clinician-collected samples were used (3.8% vs. 2.8%, p-value 0.50). Overall level of agreement was high (≥ 96.2%) in all genotype groups.

**Table 2:** HPV genotype positivity and overall agreement within HPV genotype groups between self-samples and clinician-collected samples

HPV genotype group	Positive cases		Agreement (%)	P-value^a^
	Clinician-collected sample (%)	Self-sample (%)	(95% CI)	
HPV 16/18^b^	9 (4.3%)	11 (5.2%)	97.2 (93.9–99.0)	0.69
HPV 31/33/58^c^	8 (3.8%)	6 (2.8%)	99.1 (96.6–99.9)	0.50
HPV 45/51/52^d^	6 (2.8%)	7 (3.3%)	96.7 (93.3–98.7)	1.00
HPV 35/39/68^e^	13 (6.1%)	12 (5.7%)	96.7 (93.3–98.7)	1.00
HPV 56/59/66^f^	3 (1.4%)	5 (2.3%)	96.2 (92.7–98.4)	0.73

*CI* confidence interval, *HPV* human papillomavirus

^a^McNemars test

^b^HPV16/18: HPV16 and/or HPV18 including co-infections with HPV genotypes of lesser risk (HPV 31, 33, 58, 45, 51, 52, 35, 39, 68, 56, 59 and 66)

^c^HPV31/33/58: HPV31 and/or HPV33 and/or HPV58 including co-infections with HPV genotypes of lesser risk (HPV 45, 51, 52, 35, 39, 68, 56, 59 and 66)

^d^HPV45/51/52: HPV45 and/or HPV51 and/or HPV52 including co-infections with HPV genotypes of lesser risk (HPV 35, 39, 68, 56, 59 and 66)

^e^HPV35/39/68: HPV35 and/or HPV39 and/or HPV68 including co-infections with HPV genotypes of lesser risk (HPV 56, 59 and 66)

^f^HPV56/59/66: HPV56 and/or HPV59 and/or HPV66 with no co-infections

## Discussion

### Main findings

Despite small numbers, this study found that when using the Evalyn brush and the CLART HPV assay in a referral population, self-samples detected at least as many HPV positive cases as clinician-collected samples. The self-samples detected approximately the same number of CIN2+ cases as compared to the clinician-collected samples. The same genotypes were detected in 75% (21/28) of the HPV-positive paired samples, while at least one identical genotype was found in the remaining 25% (7/28) of the paired samples.

Overall, we found that self-samples performed at least as good as clinician-collected samples with respect to HPV detection and genotype agreement.

### Strengths and limitations

The main strength of this study was the paired design in which the women acted as their own control and lowered the risk of confounding factors. In addition, we used a combination of a clinically validated self-sampling brush device [23] and a full genotyping HPV DNA assay approved for primary HPV-based cervical cancer screening [11]. Besides, an unsupervised self-sampling setting was used which may be considered an appropriate setting seen from an implementation point of view. The accuracy of HPV-testing on self-samples was expected to be independent of the HPV-results of the clinician-collected samples as the laboratory staff who handled and tested the self-samples were blinded to the clinician-collected sample results. Thus, the risk of information bias was considered low.

The main limitation was the time span between the sample sets. The time interval could have increased inequality between results from self-samples and clinician-collected samples as the 24 conflicting cases to some extent can be explained by women who cleared an existing HPV infection or acquired a new infection, or reactivation of a previous latent HPV infection [24] between sampling.

Only 19.5% of the eligible participants accepted the study invitation, which may lead to selection bias if participants and non-participants (80.5%) differed in their capacity to collect the vaginal self-sample correctly, i.e. collect a sufficient number of cervico-vaginal cells. However, as none of the self-samples was invalid for HPV testing, this scenario was considered less likely.

Ideally, histological results should have been available for all women with an HPV-positive self-sample and not only from women with an HPV-positive clinician-collected sample but this was not possible within this study population. This methodological issue was also the reason why we avoided to present the clinical accuracy of self-samples using histology as reference.

Moreover, if histological results from all samples had been gathered, we would also have been able to predict whether the false-negative and false-positive samples depended on the women clearing or acquiring infections or if the samples were inaccurate or insufficient.

While many studies have reported the dominance of HPV16 and HPV18-associated cervical cancer, there is to the best of our knowledge no clear consensus on the ranking of the next most oncogenic genotypes. For instance, HPV45 was ranked as the third most common oncogenic genotype globally by Sanjose et al. [25] and Tjalma et al. [26], but ranked as 11th for CIN3 or worse after long-term follow-up [27]. We decided to group the genotypes according to the risk of having CIN2+ among women referred to colposcopy infected with this genotype, as evidenced by Danish and Italian data [6], where HPV45 was ranked as 11th for CIN2+ and 7th for CIN3+. Based on these data, we chose to group HPV45 as middle oncogenic (group 3). Given that we had two clinician-collected and three self-collected samples that contained exclusively HPV45, a re-categorization of the groups would only have affected our results slightly and not our conclusion.

Since the study was conducted in a referral population among women already diagnosed with ASC-US, the results cannot be directly generalised to a screening population and should therefore be interpreted with caution. In addition, the results should be interpreted with caution due to the limited number of HPV positive cases.

### Interpretation and comparison with existing literature

Our study showed good concordance between vaginal self-samples and clinician-collected samples (κ: 0.63) for HPV detection (any type). Our result corresponds to the good concordance (κ: 0.70) observed in a previous study by Tranberg et al. within the same study population using the COBAS 4800 assay [15]. Our concordance using CLART was lower than the mean kappa (κ: 0.71) shown for brush devices tested with different PCR-based HPV DNA assays shown in a review by Schmeink et al. [28]. This could be explained by variations in self-sampling devices, HPV assays, laboratory protocols, and study populations as well as the time span between the samples in our study. Compared to the previous COBAS data [15], the CLART assay showed an almost similar overall agreement (88.7% for CLART and 89.2% for COBAS). With respect to genotypes, the highest agreement (99.1%) was found for HPV31/33/58 when using CLART which is somewhat higher than the overall agreement of 95.7% detected for HPV16/18 when using the COBAS assay [15]. Importantly, we found no discordance in the genotype agreement as exactly the same genotypes were discovered in 75% of the paired samples and at least one identical genotype in the remaining 25%. These results are in agreement with a Dutch study using the clinically validated full genotype GP5+/6+-LQ HPV DNA assay reporting 77% of samples with exactly the same genotypes and 23% of samples with at least one identical genotype when comparing clinician-collected samples with self-samples using the Evalyn brush device [18]. The Dutch VERA study, utilizing the Evalyn Brush and COBAS in a screening population, found a higher genotype agreement as 96.4% of sample sets had exactly the same genotypes, 0.4% had at least one identical genotype and 3.2% of the paired samples were discordant [23]. These differences may be due to different study populations and assays.

In our study, HPV infections were detected in 19.3% of the self-samples. This prevalence was higher than, but not significantly different from, the 14.8% reported by Enerly et al. and the 11.3% reported by Lam et al. which used the CLART HPV2 assay in combination with the Evalyn brush to investigate the HPV prevalence among self-samplers [29, 30]. The difference observed might be explained by different study populations (screening vs referral populations) as well as different versions of the CLART assay. The HPV prevalence in self-samples (19.3%) and clinician-collected samples (18.4%) reported in this present study, was lower but not significantly different than reported in our previous COBAS data with corresponding 24.4% and 22.1% of the samples, testing HPV-positive. Whether using more concentrated purified DNA material and/or the MagNA Pure 96 platform (Roche Diagnostics, Switzerland) for DNA extraction [29, 31] could result in higher HPV positivity rates for the CLART assay needs further evaluation.

Consistent with other studies [5, 15, 23] using different clinically validated HPV DNA assays reporting either partial, extended or full genotyping, we observed a slightly but not significantly higher HPV prevalence in the self-samples as compared to the clinician-collected samples (19.3% vs 18.4%, p-value 0.84). The higher HPV prevalence in self-samples compared to clinician-collected samples may be problematic if the HPV-positive self-samplers do not have underlying CIN2+ as this will result in false positive test results. As we found more CIN2+ among the HPV positive self-samples than among the clinician-collected samples, the use of genotype-specific referral strategies seems to be valuable. One could imagine two outcomes from a HPV-positive self-sample: 1) HPV16,18,31,33,45,51,52,58 positive women could be directly referred for colposcopy and biopsies and 2) other high-risk HPV positive women could be referred for cervical cytology-triage at the clinician or asked to repeat self-sampling. It may also be possible, that the best risk stratification strategy among HPV-positive self-samplers could be a combination of genotyping and DNA methylation profiling [32, 33].

## Conclusions

The CLART HPV4S assay performed at least as good in vaginal self-samples as in clinician-collected cervical samples with respect to both HPV detection and genotype agreement. Although further evaluation is needed, the findings suggest that full HPV genotyping based on the CLART assay can be useful when establishing HPV genotype-specific referral strategies for women tested HPV-positive by self-sampling.

## Data Availability

The dataset used in this study contains personal information and is not publicly available. An anonymised dataset is available from the corresponding author upon reasonable request and with permissions from relevant Danish Authorities.

## Abbreviations

AIS: Adenocarcinoma in situ
ASC-US: Atypical squamous cells of undetermined significance
CDR: Central Denmark Region
CFTR: Cystic fibrosis transmembrane conductance regulator
CI: Confidence interval
CIN: Cervical intraepithelial neoplasia
CIN1: Cervical intraepithelial neoplasia of grade 1
CIN2: Cervical intraepithelial neoplasia of grade 2
CIN2+: Cervical intraepithelial neoplasia of grade 2 or worse
CIN3: Cervical intraepithelial neoplasia of grade 3
GP: General practitioner
HPV: Human papillomavirus
IQR: Inter quartile ranges
PCR: Polymerase chain reaction
